# Age structure of bed bug (Heteroptera: Cimicidae) aggregations affects the nymphal feeding success

**DOI:** 10.1186/s13071-019-3659-5

**Published:** 2019-08-13

**Authors:** Ondřej Balvín, Petr Chajma, Richard Naylor

**Affiliations:** 1 Department of Ecology, Faculty of Environmental Science, Czech University of Life Sciences Prague, Kamycka 129, 165 21 Prague 6, Czech Republic; 2 The Bed Bug Foundation, Prior’s Loft, Coleford Road, Tidenham, Chepstow, Monmouthshire NP16 7JD UK

**Keywords:** Bed bug, Sub-social structure, Feeding, Aggregation

## Abstract

**Background:**

Bed bugs (Heteroptera: Cimicidae) are a group of blood-feeding ectoparasites. They mainly specialize on bats and birds, but a few species are important human pests. They exhibit several unique adaptations for their parasitic lifestyle. Among those, bed bug aggregations represent a striking example of a sub-social structure. However, their benefits for the bed bugs as well as their potential for bed bug control are largely unexplored. Young nymphs are known to disperse from the aggregations much less than older ones or adults. We therefore found possible that the aggregation age structure is connected with success in finding host and tested the effect of presence of adults on nymphal feeding success.

**Results:**

We tested the effect of presence of adults on feeding success of first-instar nymphs using an artificial feeding system. We found that presence of fed adults causes larger proportion of nymphs to feed.

**Conclusions:**

Based on our data, fed bed bugs seem to trigger the young nymphs to actively forage. Since the first instar is much less viable than later stages, our finding points to an adaptive behavior that economizes on foraging energy cost. In the context of bed bug control, knowledge on such behavior emphasizes the prevention of fed bed bugs from returning to harborages. Bed bug traps may thus be used not just as means of bed bug monitoring, but also as means of control.

**Electronic supplementary material:**

The online version of this article (10.1186/s13071-019-3659-5) contains supplementary material, which is available to authorized users.

## Background

Insects are often found to use sub-social group structures varying in temporal cohesiveness and the degree of mutual relatedness. The groups hold together for either mere parental care or cooperation of related (kin selection) or unrelated individuals. The benefits of group living are demonstrated by efficiency of either antipredatory defence or use of food sources, create striking convergent patterns in respective mechanisms among taxa [1].

Bed bugs (Heteroptera: Cimicidae) are obligatory blood-feeding ectoparasites and, at the same time, they represent a striking example of group living sub-social insects. Their life in aggregations is an important aspect, or consequence, of their very unusual ectoparasitic strategy. Unlike most ectoparasites, they are not associated with the host body for most of their lives. They also differ from free living blood-feeders, such as mosquitoes (Diptera: Culicidae) or kissing bugs (Heteroptera: Reduviidae: Triatominae), since they have found a way to avoid searching for a new host each blood meal. Their strategy consists of occupying the shelter of their host, whose body they visit only in order to feed [2] or, sometimes, disperse [2, 3]. Likely for this reason, solitarily living hosts are not a sufficiently stable food source for most bed bug species. Therefore, they evolved to feed on colonial or social vertebrates, such as bats, swallows, swifts and pigeons [4]. Importantly, specialized lineages of two (*Cimex lectularius*, *C. hemipterus*), possibly three (*Leptocimex boueti*) bat-related species have become serious pests for humans, experiencing a dramatic resurgence in past decades [5].

In the host shelter, the bed bugs create aggregations. The members of the aggregations are often highly related, depending on the population dynamics mediated by the host and the host environment [6, 7], pointing to kin selection as a possible prerequisite of their group lifestyle. The bed bug aggregations consist of all live stages and both sexes, with adults representing approximately 30% in a reproducing colony [8]. The aggregations are mediated by both airborne and contact aggregation pheromones [9]. The tendency to aggregate decreases with hunger [10].

Only two studies exist directly showing the impact of aggregations on bed bug fitness. First, bed bugs were shown to develop slower when kept alone; however, the mechanism was not described [11]. Secondly, aggregation protects the bed bugs from dehydration [12]. As pointed out by Reinhardt & Siva-Jothy [13], the implication of aggregation ecology for bed bug control has not been examined, apart from use of aggregation pheromones in bed bug traps.

We believe that age structure of an aggregation can affect the ability of bed bugs to locate the host, and, consequently, the feeding success. Wang et al. [14] found that nymphs are nine times less likely to disperse than adults, though instars were not distinguished. Naylor [15] found only older nymphal instars dispersing, while first instars remained in the refugium. As females prevail among dispersing bed bugs [2, 3, 14], their dispersal is often explained by the motivation to start a new infestation, as females are able to store sperm for weeks and therefore locate new hosts and found new infestations on their own. However, males and older nymphs disperse for long distances as well [14, 15]. At least in the nymphs, this can only be explained by nutrition demand. Although previous researchers do not agree on the maximum distance for which the bed bug is able to directionally locate a host (4–5 cm [16, 17] or 150 cm [10]), they do agree on the significance of random appetitive searching over this distance. Considering the age structure of bed bugs found to disperse, it is very likely that searching is mostly done by adults or older nymphs. Therefore, we hypothesize that host location by young nymphs depends on their older conspecifics in the refugium.

Undoubtedly, the significance of aggregation of bed bugs is yet to be explored. In our study, we aim to investigate a major aspect of bed bug life, the feeding ecology, with respect to age structure of aggregations. We tested the feeding success of first-instar nymphs in relation to presence of adult bed bugs. We aim to show that nymphs aggregating with adults benefit from enhanced feeding success, thus demonstrating a clear advantage of group living, which may explain the evolution of sociality in bed bugs.

Such knowledge on bed bug feeding ecology can also prove very useful in bed bug control as well. In case the nymphal feeding is dependent on the return of adults to the refugia, means of preventing the return should be searched for. Our study uses the common bed bug *Cimex lectularius* as a model, similarly as a vast majority of studies on bed bug biology and control.

## Methods

The study uses three population replicates; strains collected from human associated infestations in the Czech Republic in 2014 designated as “B” (Beroun, central Bohemia), “Č” (Prague, Čestmírova street) and “O” (Havířov, Ostrava region). The bed bugs were kept at 27 °C, 50% relative humidity (RH), 12:12 h light:dark regime and fed weekly in the middle of scotophase on parafilm bags [18] filled with human blood conserved in CPDA (citrate phosphate dextrose adenine conserved commercial blood purchased from Faculty Hospital Královské Vinohrady, Prague). The tubes used for both keeping the colonies and the trials were constructed from 50 ml Falcon tubes, replacing the conical bottom with a mesh, through which the bed bugs were fed. The colonies were kept on folded pieces of 80 g/m^2^ black paper and sorted weekly in order to maintain optimal breeding conditions.

To test the adult influence on nymphal feeding success, we founded groups consisting of 7 freshly-fed individuals (5 females, 2 males; an optimal combination with respect to the number of offspring to test and certainty that the females are mated). These were placed into a clean (washed with soap and alcohol) falcon tube equipped by a straight piece of paper 25 × 45 mm (half of the tube length) which was made sure to remain in contact with the mesh through the whole experiment. After one week, adults were removed from half of the groups, paying attention not to damage or lose the eggs. After two weeks, when most or all viable eggs had hatched, the groups were fed for 30 min, placing the tubes with or without adults in random order across the feeding device. Each week, only one feeding session was carried out (i.e. the maximum number of groups that fit the capacity of the feeding device, which was 16 stations). After feeding, the numbers of fed and unfed adults and nymphs were recorded. During the two weeks, some adult mortality was observed, making the total number of adults range from four to seven in tubes where adults were kept.

To test the influence of adult presence on nymphal feeding success, two generalized linear mixed effects models (GLMM) with binomial distribution and logit link function were used. The first model evaluated the nymphal feeding success (as the dependent variable) of all of the groups (regardless of adults’ removal) depending on (i) the number of adults present; (ii) the number of bed bug nymphs present; and (iii) their interaction as fixed effects. In order to account for variation among strains and possible influence of date of the experiment, (iv) strain and (v) the date were included as random intercepts. The second model evaluated the nymphal feeding success of only the groups where the adults were present for the whole duration of the experiment. By doing that, it was possible to include (vi) the proportion of fed adults, as well as (i) the number of adults present and (ii) the number of bed bug nymphs present as fixed effects. The random effects were kept the same as in the previous model: (iv) strain and (v) the date of the experiment.

To obtain *P*-values, we performed Type II Wald *χ*^2^ tests. All statistical analyses were performed in R 3.3.1 [19] using *lme4* [20] and *car* [21] packages.

## Results

**Fig. 1:**
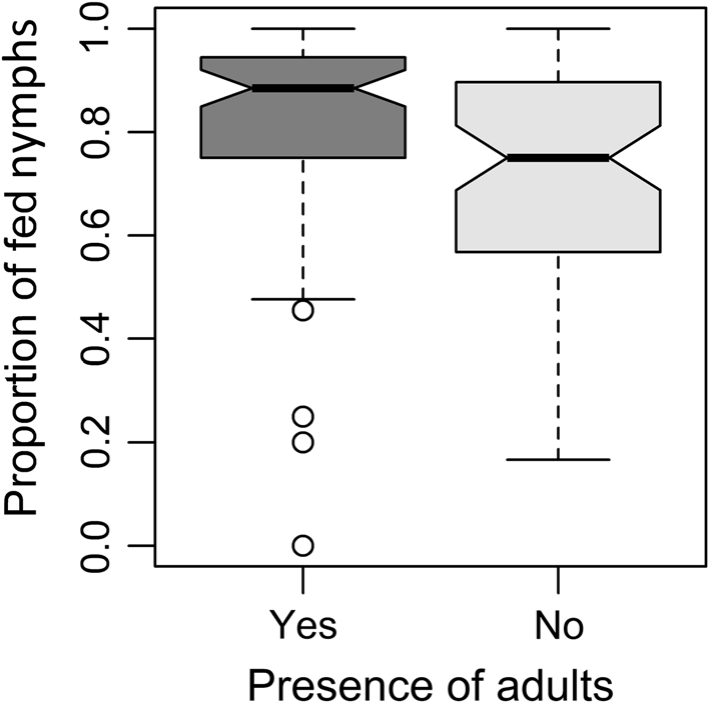
The relationship between presence of adults and proportion of fed nymphs. The notches represent the asymptotic estimate of 95 % confidence interval of median, based on [22]

When all groups were included into the model (for raw data of original measurements, see Additional file 1: Table S1), the total number of adults, i.e. their presence among nymphs, had a significant positive effect on the proportion of nymphs that have fed (*χ*^2^ = 53.40, *df* = 1, *P* < 10^−6^) (Fig. 1). Although the effect of the number of nymphs present was not significant (*χ*^2^ = 0.21, *df* = 1, *P* = 0.6466), its interaction with the total number of adults was (*χ*^2^ = 49.75, *df* = 1, *P* < 10^−6^), suggesting different responses to number of present nymphs between groups with and without adults removed.

Across the groups containing adults, 90.9% of adults fed in average. The proportion of fed adults had a significant positive effect on the proportion of nymphs that have fed (*χ*^2^ = 13.33, *df* = 1, *P* = 0.0003). The number of present nymphs had a positive significant effect as well (*χ*^2^ = 14.02, *df* = 1, *P* = 0.0002). The effect of the total number of adults, i.e. their mere presence among nymphs, was significant (*χ*^2^ = 8.30, *df* = 1, *P* = 0.0040); however, the slope was negative.

## Discussion

The result of our study supports the idea that the presence of adults in bed bug aggregations significantly increases the success of nymphs to locate and use the blood source. Noteworthy, only fed adults played such a role in our assay. In the statistical model that included only the groups with adults, the effect of proportion of fed adults was separated from the effect of total number of adults. The latter then represented the effect of unfed adults that showed to decrease the nymphal feeding success. We therefore find likely that the fed adults returning back to the harborage trigger the host location foraging effort in young nymphs. At the same time, hungry adults instead arrest the nymphs.

The first-instar nymphs are rather fragile, able to withstand the shortest period being unfed among bed bug life stages [8]. A spontaneous appetitive searching will most easily lead to their death if they fail to find a blood source. Such a trigger by freshly-fed adults can therefore effectively prevent them from wasting energy searching when the host is absent. This represents a clear benefit of group living in bed bugs.

The feeding success of nymphs was also positively affected by the total number of nymphs in the group. The effect was shown only when groups containing adults were tested. Nymphs that already fed thus appear to have a positive, presumably cumulative effect on foraging behavior of the other, still hungry nymphs. However, the effect seems to occur only when a particular proportion of nymphs manage to feed, likely in response to adult feeding. Nevertheless, such a notion implies that the size of a bed bug colony is tied with its survival and fitness through increased feeding success, similarly as the size of the colony helps the bed bugs managing humidity [12]. It thus only further highlights the importance of group living for success of bed bug parasitic lifestyle.

It is important to note that the feeding system in our assay missed some of the previously described cues that bed bugs use for locating host, i.e. carbon dioxide and host scent [4]; it only used heat. In nature, the nymphs may be therefore stimulated to forage by cues other than just the potential trigger by fed adults. Still, our assay shows the importance of interaction between fed and unfed bed bugs of different ages. Also, in the assay, the bed bugs were located only up to 45 mm from the blood source. In a natural infestation, the refugia are located rather tens or hundreds cm from the sleeping human [15] or from e.g. *Myotis myotis* clusters (O. Balvin, pers. observation). Such a direct contact clue like an encounter with a fed adult can therefore be expected to be even of a greater importance for the nymphs in nature than in our assay.

To our knowledge, our finding is the first described case of adults helping nymphs to locate a food source in ectoparasites. Only few cases have been described in insects; apart from eusocials, all of them in sub-social or gregarious species. Such behavior is known from folivores which show higher nymphal feeding success in the presence of adults [1]. Nymphs of some detritovorous cockroaches are known to track their mother using her pheromones [23] or to actively forage along their mother [24]. Apart from such cases of parental help, efficiency of foraging can be dependent on relatedness of nymphs alone, as shown in predatory mites [25]. Such behaviors belong among traits creating apparent convergence patterns in sub-social insects [1] which, similarly as eusocial species, tend to cooperate on a family basis for the sake of spreading their common genotypes. Recently, however, the aggregations of cockroaches consisting of unrelated individuals were shown to share the information on food location and benefit from group living as well [26].

Bed bugs are known to create extremely inbred populations, simply due to the fact that most infestations are founded by a single female, at least in human-related populations [6, 7, 11]. However, no group cooperation has been described in bed bugs yet. Our finding cannot be defined as cooperation either, as we have no evidence for trade-off for adults for showing the nymphs the way to the food source, apart from kin fitness. Nevertheless, it clearly represents a particular advantage of bed bug group living and accurately falls in a behavioral complex expected to develop in sub-social insects [1].

Additionally, our finding on the significance of bed bug aggregations may have an impact on bed bug control. In the case the nymphs actually rely on information on host presence provided by adult foraging, the survival of nymphs remaining in the refugia is dependent on the return of fed adults. Our results emphasize the significance of pheromone bed bug traps. These are considered to serve as bed bug monitors rather than means of bed bug eradication. However, the significance of group living that our study points to suggests that luring fed adults back to alternative locations, away from the refugia where the nymphs reside, may have significant negative implications for the feeding success and therefore survival of the nymphal stages.

## Conclusions

Our study demonstrates an advantage of a sub-social behavior in parasitic bed bugs. We show that adult bed bugs, and only fed adult bed bugs have a positive effect on feeding success of first-instar nymphs. Adults that failed to feed appear rather to arrest the nymphs from feeding. Such behavior can save the energy cost of foraging, which can substantially help the fragile nymphs to survive. Disrupting such bond between bed bug life stages may also show useful in treating bed bug infestations.

## Additional file

**Additional file 1: Table S1.** Raw data of feeding success of experimental groups.

## Data Availability

All data generated and analysed during this study are included in this published article and in Additional file 1.

## Abbreviations

CPDA: citrate phosphate dextrose adenine
GLMM: generalized linear mixed effects models
